# Supplementary material for: Disease-associated amino acid substitutions outside canonical motor motifs impair Myo1e localization and activity
Source: bioRxiv. 2026 Oct 19:2026.10.15.759198. Preprint. [Version 1] doi: 10.64898/2026.10.15.759198 (PMC13738512; doi:10.64898/2026.10.15.759198)
Supplement: Supplementary file 1 [file NIHPP2026.10.15.759198v1-supplement-1.pdf]

bioRxiv preprint doi: https://doi.org/10.64898/2026.10.15.759198; this version posted October 19, 2026. The copyright holder for this preprint (which was not certified by peer review) is the author/funder, who has granted bioRxiv a license to display the preprint in perpetuity. It is made available under a CC-BY 4.0 International license.

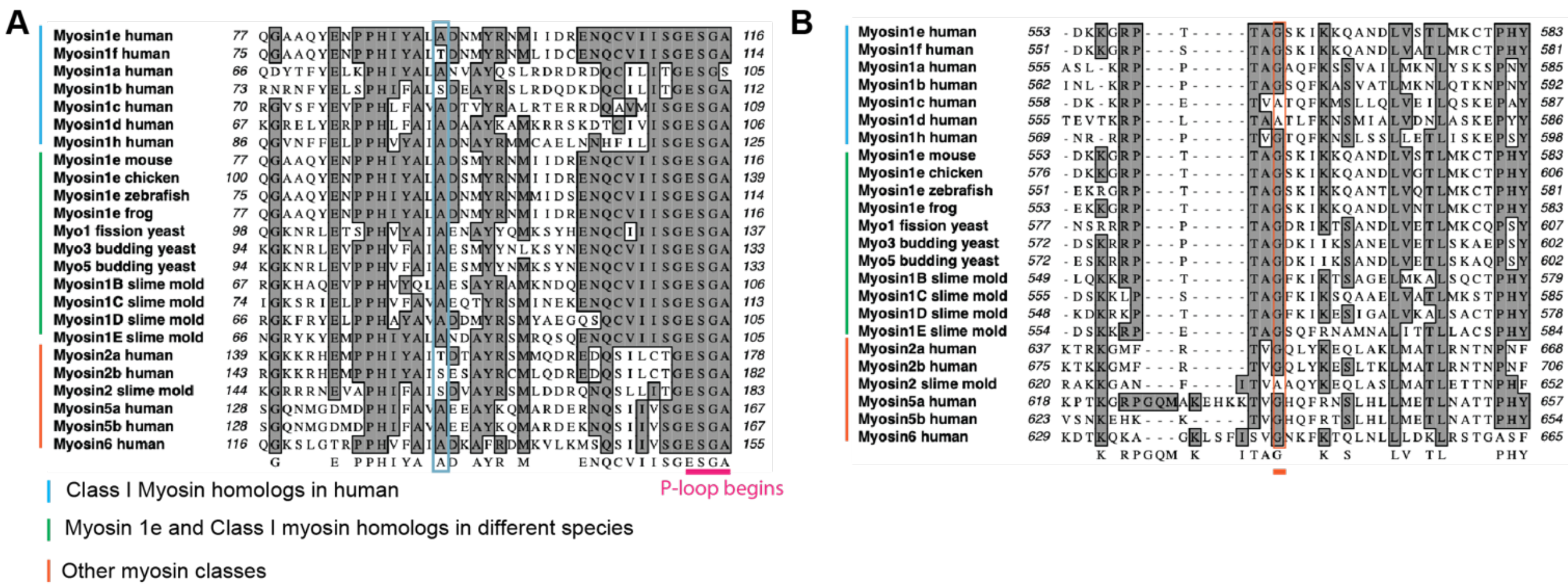

Supplemental Figure 1. Multiple sequence alignment of regions surrounding A92 (A) and G562 (B) residues. The purple box in (**A**) and the orange box in (**B**) denote A92 and G562 residues, respectively. (**A**) A92 is highly conserved across Myo1e homologs in humans, class I myosins in different species, and other myosin classes, with substitutions with polar amino acids (serine or threonine) in human Myosin 2a and Myosin 2b, Dictyostelium discoideum (slime mold) Myosin 2, Myosin 1f and Myosin 1b in humans. (**B**) G562 is also broadly conserved across Myo1e homologs, class I myosins, and non-muscle myosin, except for Myosin2 in Dictyostelium discoideum (slime mold), Myosin 1c and Myosin 1d in humans, in which the corresponding position is occupied by a non-polar amino acid, alanine.

Supplemental Table 1: Primers used for mutagenesis

Mutation type		Sequence	Length
A92E	Forward	CTATGCCCTTGAAGATAATATGTAC	25
	Reverse	ATATGTGGTGGGTTTTC	17
G562R	Forward	AACTACTGCCAGCAGCAAAATAAAGAAACAAG	32
	Reverse	GGGCGCCCTTTCTTG	15

Supplemental Table 2: NCBI reference sequence accession number

Protein	Species	Accession number
Myo1e	Human (Homo sapiens)	NP_004989.2
Myo1e	Mouse (Mus musculus)	NP_851417.2
Myo1e	Zebrafish (Danio rerio)	NP_956930.1
Myo1b	Human (Homo sapiens)	NP_036355.2
Myo1f	Human (Homo sapiens)	CAC83948.1
Myo1	Fission yeast (Schizosaccharomyces pombe)	NP_595402.1

bioRxiv preprint doi: https://doi.org/10.64898/2026.10.15.759198; this version posted October 19, 2026. The copyright holder for this preprint (which was not certified by peer review) is the author/funder, who has granted bioRxiv a license to display the preprint in perpetuity. It is made available under a CC-BY 4.0 International license.

Myo2a	Human (Homo sapiens)	NP_002464.1
Myo1B	Slime mold (Dictyostelium discoideum)	XP_636382.1
Myo5a	Human (Homo sapiens)	NP_000250.3
Myo6	Human (Homo sapiens)	NP_004990.3
Myo1a	Human (Homo sapiens)	NP_001242970.1
Myo1b	Human (Homo sapiens)	NP_036355.2
Myo1c	Human (Homo sapiens)	NP_203693.3
Myo1d	Human (Homo sapiens)	NP_056009.1
Myo1h	Human (Homo sapiens)	NP_001094891.4
Myo1e	Chicken (Gallus gallus)	XP_413782.4
Myo1e	African frog (Xenopus laevis)	XP_018108508.1
Myo3	Budding yeast (Saccharomyces cerevisiae)	NP_012793.2
Myo5	Budding yeast (Saccharomyces cerevisiae)	NP_013827.1
Myo1C	Slime mold (Dictyostelium discoideum)	XP_643060.1
Myo1D	Slime mold (Dictyostelium discoideum)	XP_643446.1
Myo1E	Slime mold (Dictyostelium discoideum)	XP_636580.1
Myo2b	Human (Homo sapiens)	NP_001242941.1
Myo2	Slime mold (Dictyostelium discoideum)	XP_637740.1
Myo5b	Human (Homo sapiens)	NP_001073936.1

bioRxiv preprint doi: https://doi.org/10.64898/2026.10.15.759198; this version posted October 19, 2026. The copyright holder for this preprint (which was not certified by peer review) is the author/funder, who has granted bioRxiv a license to display the preprint in perpetuity. It is made available under a CC-BY 4.0 International license.

Supplemental Movie 1. In vitro motility of wild-type (WT) Myo1e (1 μM) in motility buffer. Time-lapse images were acquired at 10-second intervals for up to 10 minutes.

Supplemental Movie 2. In vitro motility of the Myo1e^{A92E} mutant under the same experimental and imaging conditions as Supplemental Movie 1.

Supplemental Movie 3. In vitro motility of the Myo1e^{G562R} mutant under the same experimental and imaging conditions as Supplemental Movie 1.
